# Modelling epilepsy in the mouse: challenges and solutions

**DOI:** 10.1242/dmm.047449

**Published:** 2021-03-01

**Authors:** Grant F. Marshall, Alfredo Gonzalez-Sulser, Catherine M. Abbott

**Affiliations:** 1 Centre for Genomic and Experimental Medicine, MRC Institute of Genetics and Molecular Medicine, University of Edinburgh, Western General Hospital, Crewe Road, Edinburgh EH4 2XU, UK; 2 Simons Initiative for the Developing Brain, University of Edinburgh, Edinburgh EH8 9XD, UK; 3 Centre for Discovery Brain Sciences, 1 George Square, University of Edinburgh, Edinburgh EH8 9XD, UK

**Keywords:** Mouse, Epilepsy, Induced seizures, Genetic models

## Abstract

In most mouse models of disease, the outward manifestation of a disorder can be measured easily, can be assessed with a trivial test such as hind limb clasping, or can even be observed simply by comparing the gross morphological characteristics of mutant and wild-type littermates. But what if we are trying to model a disorder with a phenotype that appears only sporadically and briefly, like epileptic seizures? The purpose of this Review is to highlight the challenges of modelling epilepsy, in which the most obvious manifestation of the disorder, seizures, occurs only intermittently, possibly very rarely and often at times when the mice are not under direct observation. Over time, researchers have developed a number of ways in which to overcome these challenges, each with their own advantages and disadvantages. In this Review, we describe the genetics of epilepsy and the ways in which genetically altered mouse models have been used. We also discuss the use of induced models in which seizures are brought about by artificial stimulation to the brain of wild-type animals, and conclude with the ways these different approaches could be used to develop a wider range of anti-seizure medications that could benefit larger patient populations.

## Abstract (precis)

**Summary:** This Review discusses the challenges of modelling epilepsy in mice, a condition in which the outward manifestation of the disorder appears only sporadically, and reviews possible solutions encompassing both genetic and induced models.

## Introduction: what is epilepsy?

Epilepsy is one of the most common neurological conditions worldwide, with an incidence of just under one in every hundred people in the UK, and a peak in occurrence in very young and very old people (Beghi and Giussani, 2018). It is a condition that results from disruptions in network activity in the brain that manifests as seizures. Although anti-seizure drugs [ASDs; also known as anti-epileptic drugs (AEDs); see Glossary, Box 1] can be used to treat patients, these have very variable efficacies and are palliative, as there is no cure for epilepsy.

**Box 1. Glossary**

**Anti-seizure drug (ASD**): medication to treat seizures in people with epilepsy; these drugs are also known as anti-epileptic drugs (AEDs).

**Electrographic seizures**: seizures that are only identifiable by monitoring with electroencephalography (EEG), with no outwardly observable signs.

**Epileptic encephalopathies**: disorders in which epileptic activity in the maturing brain contributes to severe progressive cognitive and behavioural impairments beyond that predicted from the underlying pathology.

**Epileptiform:** specific discharges in the brain, usually detected via EEG.

**Interictal:** the periods between seizures in a patient or animal model.

**Lennox–Gastaut syndrome:** a complex, rare and severe childhood-onset epilepsy characterised by multiple and concurrent seizure types and cognitive dysfunction.

**Kindling**: repeated induction of seizure activity leading to progressively more severe seizures in terms of behaviour and duration.

**Overt, convulsive or behavioural seizures**: visually obvious seizures that can be accurately and reliably detected without the need for EEG. Examples include tonic-clonic seizures, convulsions and wild running.

**Maximal electroshock seizure test:** a highly effective test for detecting novel ASDs in which an electrical pulse is given either through mouth or scalp electrodes to induce generalised tonic-clonic seizures.

**Myoclonic jerk**: a brief and involuntary muscle spasm with irregular muscle twitching.

**Racine stages**: a widely used observational system for categorising seizure severity in animal models, originally proposed in the 1970s.

**Sudden death in epilepsy (SUDEP)**: when an individual with epilepsy dies suddenly and unexpectedly with or without evidence of a seizure and where no other obvious cause of death can be found.

**Temporal lobe epilepsy (TLE**): is characterised by focal seizures that originate in the temporal lobe of the brain. One-third of patients with TLE have drug-resistant seizures.

There are many different types of seizures with different origins and frequencies. The three main categories of epilepsy, as defined by the International League Against Epilepsy (ILAE), are based on the origin of onset: seizures of generalised onset involve large bilateral brain areas, focal onset seizures arise in a specific region on one side of the brain, and the remaining category comprises seizures of unknown onset. Sudden death in epilepsy (SUDEP; Box 1), when an individual dies during or after a seizure and often while they are sleeping, occurs in one in 1000 people with epilepsy who are otherwise healthy each year.

**Fig. 1.**
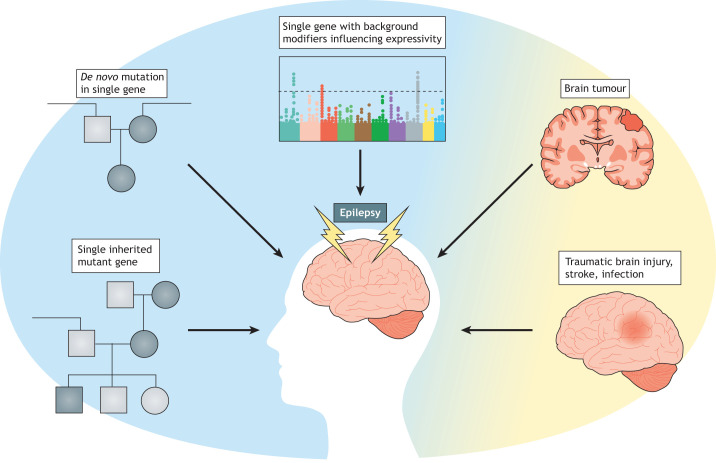
**The common causes of epilepsy, from purely genetic (blue) to purely environmental (yellow).**

The causes of epilepsy range from purely environmental, such as traumatic brain injury, through multifactorial causes like brain tumours, to purely genetic, like inherited single-gene disorders or *de novo* heterozygous dominant mutations (Fig. 1). Epilepsy frequently starts in later life and is usually associated with underlying cardiovascular or neurodegenerative disease or with physical insults such as head injury. The worldwide incidence in over 65s is 240 in every 100,000 (Liu et al., 2016). However, epilepsy is more common in childhood, with a particularly high incidence in children under 2 years old (Wirrell et al., 2011). Multiple monogenic epileptic encephalopathies (Box 1) have been identified (for a detailed review, see McTague et al., 2016). Importantly, in these cases, the epileptiform activity (Box 1) may, in itself, be harmful to development and contributes to additional cognitive and behavioural impairment.

Over a hundred single-gene causes of epilepsy have now been identified, with no one gene underlying more than 1% of total cases (reviewed in Helbig and Ellis, 2020). Initially, many of these were inherited mutations in genes encoding ion channels. However, the advent of exome and later whole-genome sequencing fostered the discovery of many new epilepsy genes. Trio sequencing, which entails sequencing of the exomes of both parents and the affected child, has been a particularly successful approach for pinpointing mutations that have arisen *de novo*. Without sequencing, these cases would, of course, not have been diagnosed as genetic as they manifest sporadically rather than running in families. Many of the newly discovered epilepsy genes overlap with those found to be causative of autism, intellectual disability and other neurodevelopmental disorders. These genes encode not just ion channels, but proteins with roles ranging from synaptic vesicle recycling (Dhindsa et al., 2015) and protein synthesis (de Ligt et al., 2012; Lam et al., 2016) to basic metabolic functions like glucose transport (Seidner et al., 1998). The underlying mechanisms by which these newly identified mutations result in network disorders often remain to be identified, depending on the function of the gene and the ease with which mutations can be modelled. However, there are some notable exceptions for which different mutations in a single gene can lead to either a loss or gain of function, depending on whether the mutation is a nonsense or deletion, as opposed to a missense, mutation. This distinction can be very important for therapy, as discussed in more detail below.

How then, with this complex picture, can we best model epilepsy in animals? Although we limit the discussions in this Review to modelling epilepsy in mice, researchers also use other model systems with their own advantages and disadvantages (Box 2).

**Box 2. Genetically manipulable model systems for epilepsy beyond the mouse**

**iPSCs**

Human induced pluripotent stem cells (iPSCs) were first generated in 2007 from fibroblasts and can now be efficiently generated from patients with neurological disorders and healthy controls (Kogut et al., 2018; Takahashi et al., 2007). Patient-derived iPSC lines are useful for modelling disorders with complex genetic architecture, which would be impractical to model in whole animals. Otherwise, control iPSC lines can also be edited using tools such as CRISPR/Cas9 to recapitulate mutations seen in monogenic disorders (Eggenschwiler et al., 2016).

Diverse neuronal and non-neuronal cell types can be differentiated from iPSCs (Wiegand and Banerjee, 2019), enabling mechanistic studies and drug screening in human tissue. The effect of candidate drugs on spontaneous neural activity can be assessed at high throughput using multi-electrode array setups (Tidball and Parent, 2016). Aside from differences in the differentiation potential of iPSCs from different donors (Kyttälä et al., 2016), human iPSC-derived neurons are slow to fully differentiate (Nicholas et al., 2013) and slow to develop mature electrophysiology (Prè et al., 2014). Moreover, human neuronal cultures do not consistently develop synchronous network activity unless co-cultured with human or murine astrocytes (Kuijlaars et al., 2016), limiting their usefulness for modelling network disturbances in epilepsy.

However, recent advances in three-dimensional culture techniques and differentiation methods have allowed the generation of cerebral organoids from single iPSC lines. These organoids comprise diverse neuronal and glial cell types, which show limited self-organisation and exhibit spontaneous, synchronised neural activity (Izsak et al., 2019; Trujillo et al., 2019; Zafeiriou et al., 2020), meaning that they could eventually model both network-level and cell-autonomous defects in epilepsy.

***Caenorhabditis*** ***elegans***

The roundworm *C. elegans* has a relatively simple but exceptionally well-characterised nervous system consisting of exactly 302 neurons (in the adult hermaphrodite) with a highly stereotyped organisation (Chatterjee and Sinha, 2007). *C. elegans* neurons are morphologically similar to mammalian neurons and use conserved neurotransmitters, although there are important physiological differences – *C. elegans* neurons have ion channels but they do not have voltage-gated sodium channels (Bargmann, 1998; Risley et al., 2016). Nevertheless, it has been estimated that over 80% of protein-coding *C. elegans* genes have human homologues (Lai et al., 2000). Seizure-like activity in *C. elegans* typically includes convulsions or head bobbing. For example, worms with loss-of-function mutations in GABA_A_ receptors are susceptible to pentylenetetrazol (PTZ)-induced head-bobbing convulsions (Williams et al., 2004; Wong et al., 2018). Head bobbing can also be induced in *C. elegans* by increasing their temperature above 26°C (Pandey et al., 2010).

*C. elegans* are cheap to maintain, reproduce rapidly, and can be treated with drugs via food (Kaletta and Hengartner, 2006). Combined with easily induced and assayed seizures, these features make them suitable for high-throughput drug screening (Wong et al., 2018). Electrophysiological recording is also possible in *C. elegans* (Goodman et al., 2012; Stawicki et al., 2013).

***Drosophila***

Around three-quarters of human disease genes have orthologues in *Drosophila* (Bier, 2005; Pandey and Nichols, 2011). Researchers using *Drosophila* as a disease model can exploit a wide variety of assays and sophisticated genetic tools to understand gene function and disease mechanisms (Ugur et al., 2016).

*Drosophila* have been used extensively as epilepsy models, with a number of seizure-susceptible mutants available (Parker et al., 2011a). For example, *bang senseless* is a gain-of-function mutation of the *paralytic* gene, which encodes a voltage-gated-sodium channel subunit. *para^bss1^* is associated with a stereotyped sequence of spasms and paralysis in response to various mechanical stimuli (Parker et al., 2011b). Mutations in voltage-gated sodium channels also cause seizures and epilepsy in humans (Feng et al., 2019), and several of these mutations cause seizures in *Drosophila* when knocked into *paralytic* (Kroll et al., 2015b). Seizures in *para^bss1^* flies can be partially suppressed with anti-seizure drugs (ASDs) or a ketogenic diet (Radlicz et al., 2019; Reynolds et al., 2004), suggesting shared underlying pathophysiology with patients. Furthermore, several *Drosophila* mutants have been identified that suppress seizures in *para^bss1^* flies (Kroll et al., 2015b; Saras and Tanouye, 2016). The identification of seizure suppressor or enhancer mutations in *Drosophila* can reveal novel therapeutic avenues.

*Drosophila* have low maintenance costs and a rapid life cycle, and can be administered with drugs via food, making them suitable for high-throughput drug screening. Moreover, electrophysiological recording and stimulation are routinely performed at various stages of the life cycle (Kroll et al., 2015a; Parker et al., 2011b; Ugur et al., 2016).

**Zebrafish**

Over the past decade, zebrafish have surged in popularity as models of human neurological disorders (Fontana et al., 2018). It has been estimated that 76% of human disease genes have a zebrafish orthologue (Howe et al., 2013), and both larvae and adults are susceptible to seizures. Seizures can be elicited using convulsants such as PTZ or kainic acid, or by genetic manipulation, and typically involve hyperactive swimming followed by loss of posture (Afrikanova et al., 2013; Baraban et al., 2013). Electrographic seizures can be detected using EEG, which has been developed for use in both larval (Lee et al., 2020) and adult (Cho et al., 2017) forms. Seizure activity can also be directly visualised in the transparent larvae using transgenic reporters (Burrows et al., 2020).

Zebrafish have high fecundity and are relatively cheap to maintain (Avdesh et al., 2012). The larvae, arrayed in microtitre plates and video recorded, can be used for high-throughput ASD screening (Baraban et al., 2013; Griffin et al., 2016), with candidate drugs simply added to the water. For example, a zebrafish model of Dravet syndrome (with mutations in the *SCN1A* homologue *scn1lab*) shows face validity for behavioural and electrographic seizures. Drug screening using this model identified clemizole as a potential treatment for patients (Baraban et al., 2013).

**Rats**

As well as having similar gross brain anatomy, rats possess neural circuits and patterns of network activity that are highly homologous to those in humans (Heilbronner et al., 2016; Lu et al., 2012). Around three-quarters of human disease genes have rat orthologues, and genes associated with neurological disease are particularly well conserved (Huang et al., 2004). Before the development of programmable endonucleases, the transgenic tools available for rats were lagging behind the more sophisticated tools available for mice. However, transgenesis and site-specific editing are now equally feasible in rats (Ellenbroek and Youn, 2016; Guan et al., 2014; Gurumurthy and Lloyd, 2019).

Rats have been extensively studied and are highly informative as acute seizure models, and as kindling models, and have been instrumental for ASD screening (Löscher, 2017). At present, the best-characterised genetic rat epilepsy models are inbred strains carrying spontaneous mutations, such the GAERS and WAG/Rij strains (Danober et al., 1998). However, rats with clinically relevant mutations have also been studied (Ohmori et al., 2020). Diverse EEG setups have been developed for both tethered (Medlej et al., 2019) and wireless (McGuire et al., 2019) EEG in rats, and *ex vivo* brain slice preparations can be used to study seizure susceptibility (Accardi et al., 2018) and epileptogenesis (Dyhrfjeld-Johnsen et al., 2010), and to screen ASDs (Hill et al., 2010).

## Modelling epilepsy

In order to rationally design and test novel treatments for epilepsy, researchers require whole-animal models that capture the relevant human pathology. Animal models must have construct validity, recapitulating the aetiology of the human disorder within a homologous neurobiological context; they must have face validity, exhibiting the same or similar phenotypes as patients; and they should ultimately have predictive validity, only responding to treatments that are effective in patients with the modelled disorder (Garner, 2014; Willner, 1984). Such models provide an indirect window into human pathology, allowing us to identify novel drug targets and to test the efficacy of therapies through preclinical trials.

Although there are fundamental differences between human and mouse neurobiology at all levels of organisation (Hodge et al., 2019), mice nevertheless represent strong candidate organisms for modelling human neurogenetic disorders. Over 99% of mouse genes have human homologues (Mouse Genome Sequencing Consortium et al., 2002), and, within the brain, the spatial pattern of gene expression is at least grossly conserved (Strand et al., 2007). In terms of epilepsy, focal mouse models of temporal lobe epilepsy (TLE; Box 1) mimic patients, in that ictal seizures emerge from the hippocampal formation and then spread to the rest of the brain (Riban et al., 2002). Similarly, cortical and thalamic structures are responsible for generalised seizures in both humans and mouse models (Cao et al., 2020).

**Fig. 2.**
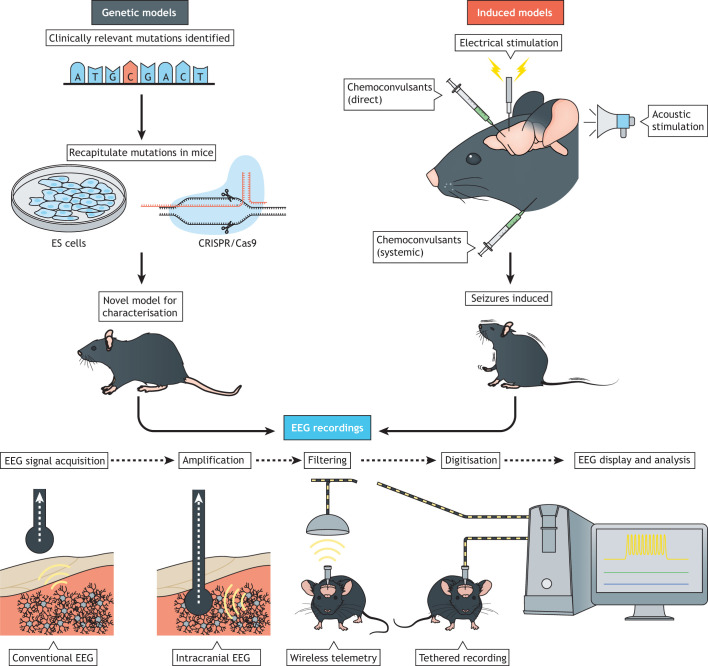
**Generation and characterisation of rodent epilepsy models.** Genetic models are generated by knockout or knock-in mutagenesis. In induced models, seizures are typically elicited by chemical, electrical or acoustic stimulation of the brain. In both model types, the seizures are the key phenotypic readout and can be accurately and reliably detected using electroencephalography (EEG). Rodent EEG begins with the installation of electrodes or electrode arrays on the skull surface in the case of conventional EEG or beneath the skull for intracranial EEG or electrocorticography. Intracranial electrodes may be positioned at the brain surface or at a higher depth. Synchronised neuronal activity near the electrodes generates the EEG signal that undergoes various stages of amplification, filtering and digitisation before finally being displayed (Moyer et al., 2017). EEG signals may be transmitted from the animal to the computer via a cable (tethered recording) or wirelessly (wireless telemetry). ES cells, embryonic stem cells.

Mouse models of epilepsy can be divided into genetic and induced (Fig. 2). In genetic models, genetic lesions (or spontaneous mutations) result in the emergence of spontaneous seizures. In induced models, chemical, electrical or acoustic stimulation result in either acute or chronic seizures, depending on the induction method and protocol.

### Genetic models

The development of programmable endonuclease-based genome editing such as CRISPR/Cas9 has significantly streamlined the generation of clinically relevant genetic mouse models (Gurumurthy and Lloyd, 2019). Of course, if we want to model genetic epilepsies, CRISPR/Cas9 enables us to generate models of specific missense mutations, as well as complete knockouts, with relative ease. These models are essential for understanding the underlying molecular mechanisms and phenotypic consequences of specific mutations, and can even be used as additional support where variants have been discovered in patients but have not yet been definitively shown to be causative. Genetic mouse models are also invaluable for studying the behavioural outcomes of network disruptions in early development, including the possibility of age-dependent reversibility, and are necessary for testing any gene-specific therapies, whether precision medicine-based therapeutics or gene therapy. There are, of course, challenges posed by the presence or absence of modifier loci in different inbred mouse strains, as discussed below. Similarly, extrapolating results from single-gene models on inbred backgrounds to human cases with highly variable genetic backgrounds will not always be straightforward, but is likely to be more successful in epileptic encephalopathies resulting from highly penetrant mutations in single genes.

The genetic background of mouse models of epilepsy, whether genetic or induced, can of course vary. This potential confounder needs to be considered when comparing results from different studies, even those using mice with apparently the same inbred strain background. However, these phenotypic differences can also be manipulated to identify genetic modifiers, and to modulate seizure severity to suit the purpose of the experiment. For example, the *Scn2a^Q54^* model displays different stage of onset of seizures, seizure frequency and survival, depending on whether the mutation is on a C57BL/6J or SJL/J background. These differences were exploited to identify *Cacna1g* as an epilepsy modifier gene (Calhoun et al., 2016). Similarly, SJL/J mice with an *Scn8a^N1768D^* mutation modelling an epileptic encephalopathy mutation seen in patients have later seizure onset and increased survival in comparison to mice with the same mutation on a C57BL/6J background. Intercross experiments showed that a hypomorphic mutation of *Gabra2* in the C57BL6/J strain increased seizure severity, suggesting that upregulation of the *Gabra2* product could be a future therapeutic strategy (Yu et al., 2020). Mouse models of different mutations in *Scn8a* (Table 1) have also been used for preclinical testing of drugs that have moved into clinical trials (Meisler, 2019).

**Table 1.**
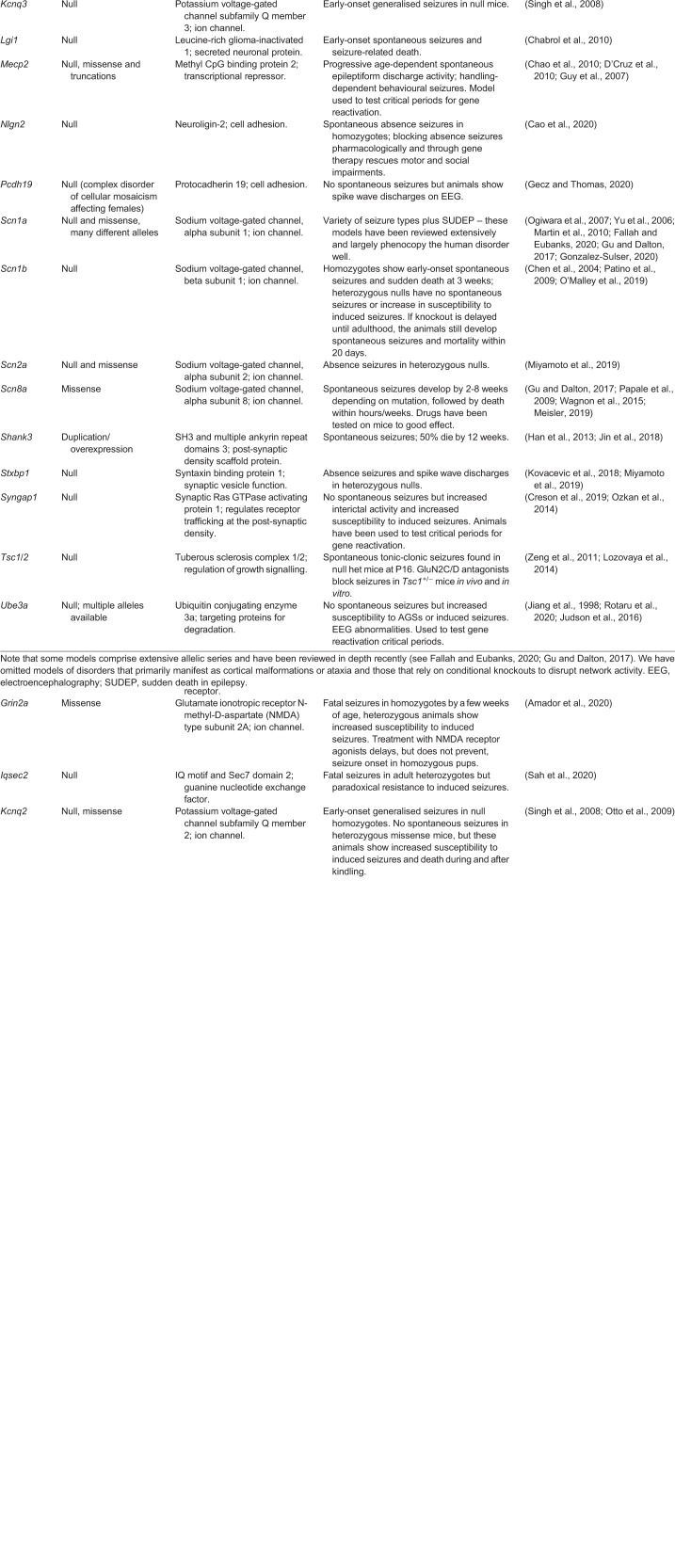
**Selected validated models of genetic epilepsies**

Importantly, genetic models can also be used to establish whether mutations operate through a gain- or loss-of-function mechanism, which can, in turn, provide crucial context for determining the appropriate therapeutic approach. There are some notable cases where, for example, different mutations in a single gene can lead to either loss or gain of function, depending on whether the mutation is a nonsense or deletion, as opposed to a missense, mutation (Davies et al., 2020; Quraishi et al., 2020). This distinction can be very important for therapy. In a recent striking example, researchers found that epilepsy resulting from mutations in *SCN2A* usually had an onset at less than 3 months of age, when the mutations were missense, and that these cases responded well to sodium channel blockers. By contrast, cases with disease onset at over 3 months generally had truncating loss-of-function mutations and failed to respond to sodium channel-blocking drugs, illustrating the importance of understanding the underlying mode of action even within a single gene (Wolff et al., 2017).

Although genetic models are clearly more useful for studying the molecular mechanisms of specific epilepsies and for generating targeted therapies, their value as preclinical models can be limited by their seizure phenotypes (or lack thereof). Later, we will discuss the limitations of genetic mouse epilepsy models in terms of seizure manifestation, before discussing how these disadvantages may be overcome.

### Induced models

Induced models of epilepsy, in which otherwise healthy animals are made to develop seizures either by exposure to a chemical compound or to electrical stimulation (Fig. 2), have been utilised for over 75 years. They have been instrumental in the identification and development of treatments (Bialer et al., 2013, 2015, 2017, 2018; Fallah and Eubanks, 2020; Löscher, 2011, 2017). Electrical shock or systemic or intracerebral injection of a convulsant compound can trigger frequent and severe seizures in mice, making the phenotypes of these models relatively easy to visually quantify while also allowing for large throughput. From the antiepileptic properties of trimethadione in 1945, which eventually became a standard treatment for absence seizures, which present outwardly as a sudden behavioural arrest (Everett and Richards, 1945), to the maximal electroshock seizure test (Box 1; Toman et al., 1946), which is still the most commonly used initial readout for screens of new ASDs, induced mouse models have played a crucial role in the discovery of many of the major epilepsy treatments in use today (Löscher, 2017).

In so-called kindling (Box 1) protocols, researchers deliver electrical (Leech and McIntyre, 1976) or chemical (Schmidt, 1987) insults on multiple occasions. Once the animals are ‘kindled’, seizures can be elicited on demand and novel treatments can be tested. Similarly, quantifiable motor seizures can be generated acutely by a systemic chemical injection of pilocarpine (Cavalheiro, 1995), kainate (Lévesque and Avoli, 2013) or pentylenetetrazole (Karler et al., 1989). In chronic epilepsy models, neuronal damage and reorganisation of connections lead to the emergence of spontaneously occurring seizures, which better mimic clinical epilepsy, as the seizures can occur at any time. For example, in the systemic pilocarpine (Cavalheiro, 1995) and intrahippocampal kainate (Riban et al., 2002) injection models, an initial insult leads to a status epilepticus event that eventually subsides and is followed by a refractory period lasting days to weeks. This coincides with the emergence of hippocampal sclerosis and spontaneous seizures. Furthermore, some ASDs are ineffective in these models, mimicking TLE, which is often difficult to treat. Induced models are therefore useful platforms for discovering new therapeutic strategies that are able to block intractable seizures (Klein et al., 2015; Lévesque and Avoli, 2013).

### Challenges in finding new treatments

Despite progress in developing novel ASDs, a substantial proportion of epileptic patients have drug-resistant epilepsy, for which medication cannot manage the seizures. Under ILAE definitions, the prevalence of drug resistance has been estimated to range from 17% to 33% of all epilepsy cases (Kwan et al., 2010; Ramos-Lizana et al., 2012; Téllez-Zenteno et al., 2014). Large screening consortia, such as the Epilepsy Therapy Screening Program, are now utilizing the above-discussed chronic and kindling models, in which conventional ASDs do not block seizures, to discover novel treatments for drug-resistant epilepsy (Löscher, 2017). However, these models are more relevant to adult TLE, for which disease mechanisms are likely to be very different from those of genetic epilepsy syndromes such as epileptic encephalopathies. As these syndromes often display drug-resistant seizures in association with intellectual disability (Zhang et al., 2017), researchers face the challenge of developing treatment for drug-resistant epilepsies of vastly varying aetiologies.

Induced models can be relied upon to exhibit acute or chronic seizures ‘on demand’ and therefore have high face validity, but their relevance to human epilepsies remains contentious. One may contend that, even though they trigger repeated seizures, chemical or electrical induction are not genuine causes of human epilepsy and will therefore fail to recreate the precise cellular and molecular pathology of any bona fide aetiology, limiting our ability to extrapolate findings to humans (Bertram, 2007; Löscher, 2011). Conversely, genetic models can precisely recapitulate the genetic mutations that cause epilepsy in patients and thus have high construct validity. Although it is clear that researchers need both induced and genetic models for maximal drug development efficiency, genetic models are likely to become increasingly important for developing properly targeted therapies. Indeed, the concept of precision medicine has had some notable successes in epilepsy, albeit mainly in the field of metabolic epilepsies. The most striking example of this is seen in GLUT1 deficiency, where mutations in the *SLC2A1* gene, which encodes a glucose transporter in the blood brain barrier (GLUT1), lead to low glucose in the cerebrospinal fluid, resulting in seizures and developmental delay (Seidner et al., 1998). Treating affected children with a ketogenic diet so that their neurons use ketone bodies as an energy source results in good seizure control (Fujii et al., 2016). This progress in precision medicine suggests that the availability of genetically altered mouse lines recapitulating specific clinical mutations could lead to the development of properly targeted, individualised therapies for groups of patients (Helbig and Ellis, 2020).

## Problems of two sorts with genetic mouse models

In the field of epilepsy research, the major challenge no longer lies in the generation of genetic models but in assessing their face validity: do these mutant mice have seizures and, if so, how frequent and severe are they?

As in humans, seizures in mice are typically detected and classified using a combination of electroencephalography (EEG) and behavioural signs, such as convulsions or myoclonic jerks (Box 1). Although all seizures involve epileptiform brain activity, not all seizures have obvious behavioural manifestations. For specific models or classes of seizure, ordinal scales such as the classical Racine stages (Box 1) have been developed to measure seizure severity (Chung et al., 2009; Racine, 1972; Singh et al., 2008). The duration of seizures can also be used as a measure of severity (Wu et al., 2020).

Frequent, overt (Box 1), but non-fatal, seizures represent ideal face-valid epileptic phenotypes: not only are they unambiguous, but they represent a direct and easily assayed readout for whether experimental treatment attempts have been effective in reducing seizure frequency or severity. Unfortunately, however, many genetic mouse models of epilepsy have seizure phenotypes that are not so conveniently assayed. In general, there are problems of two sorts: seizures that are difficult to measure and seizures that are overly severe and fatal.

### Problems of the first sort: seizures that are difficult to measure

Researchers must reliably detect and score seizures in order to obtain an accurate baseline against which to compare animals receiving experimental treatments. However, many models have relatively rare seizures, making accurate quantification difficult without continuous, long-term EEG-video recording. For example, there are many genetic mouse models in which spontaneous seizures occur only once per week or less (Kash et al., 1997; Malas et al., 2003; McColl et al., 2006; Singh et al., 2008). In some models, seizures are spread unevenly over time. In the widely used A/J (The Jackson Laboratory) mouse strain, seizures occurred mostly during sleep (Strohl et al., 2007), in common with many epilepsy patients (Chokroverty and Nobili, 2017). Other models only have spontaneous seizures within certain age ranges. For example, *Tsc1*^+/−^ mice develop frequent, spontaneous seizures at post-natal day (P)9 that resolve by 3 weeks of age (Gataullina et al., 2016; Lozovaya et al., 2014). By contrast, *Pum2*-deficient mice do not develop spontaneous seizures until 5 months of age (Follwaczny et al., 2017). The timeline for spontaneous seizure emergence can also differ substantially between patients and models. For example, mouse models of CDKL5 deficiency disorder have failed to exhibit the early-onset spontaneous seizures that occur in human patients (Amendola et al., 2014; Fehr et al., 2016; Okuda et al., 2017). Instead, spontaneous seizures in these models emerge well into adulthood at around 10 months of age (Mulcahey et al., 2020).

Aside from seizure rarity, many models exhibit non-convulsive electrographic seizures (Box 1) that may be difficult to detect or score. This is especially true for models with absence seizures (Chung et al., 2009; Jarre et al., 2017; Tan et al., 2007). The behavioural arrests typical of these seizures are often frequent but are inherently difficult to quantify without EEG recordings (Chung et al., 2009; Jarre et al., 2017). Other subtle seizures such as brief myoclonic jerks may also prove difficult to monitor. In one study of *Fosb*-null mice, sudden behavioural arrests and myoclonic jerks were simply not recorded owing to the inherent difficulty in quantifying them (Yutsudo et al., 2013).

### Problems of the second sort: severe, fatal seizures

In contrast to models with rare or subtle seizures, many genetic epilepsy models suffer from severe but fatal seizures. Typically, such models show a sudden spike in mortality concomitant with the onset of spontaneous seizures in early life, resulting in a dramatically reduced life expectancy (Bunton-Stasyshyn et al., 2019; Chung et al., 2009; Kerjan et al., 2009; Meikle et al., 2007; Zhang et al., 2016). For example, *Dcx*; *Dclk2*-null mice show increased mortality after the onset of spontaneous seizures around P16, with over 90% of mice dying by 5 months of age (Kerjan et al., 2009). This limits the translational value of such genetic models as longitudinal studies are not possible. It is also difficult to ethically justify the use of sudden death as an outcome measure for experimental treatments. Furthermore, as many of these models die after a limited number of seizures, some after a single seizure (Kerjan et al., 2009; Meikle et al., 2007), they are arguably less relevant for modelling epilepsy than as models for SUDEP (Moore et al., 2014; Wagnon et al., 2015).

### Can we get around the seizure quantification challenges in genetic models?

For researchers who have developed genetic models but have failed to detect spontaneous seizures, the question is whether the model has problems of the first sort – seizures that are difficult to measure – or whether the model simply has no face-valid phenotype. Answering this question is of key translational importance. Face validity does not only mean that relevant readouts are available for preclinical studies; it ultimately reflects the construct validity of the model, which extends beyond whether an orthologous gene exists. For example, a study has recently shown that mice recapitulating the D252H missense mutation in neuronal translation-elongation factor eEF1A2, which is associated with neurodevelopmental disorders and late childhood epilepsy, do not show face validity for spontaneous seizures or neurodevelopmental deficits, despite the fact that human and murine eEF1A2 are almost identical (Davies et al., 2020). These mice were not studied beyond 1 year of age, and it is possible that spontaneous seizures may have developed later, in line with the late onset in children. Nevertheless, their apparent lack of face validity demonstrates how recapitulating clinically relevant mutations in orthologous genes is not always sufficient to generate a face-valid disease model. Only if the biochemical milieu of the gene products is highly conserved between mice and humans, and only if pathological changes occur within a homologous developmental and neurobiological context, will human epileptogenic mutations also be epileptogenic in mice (Belzung and Lemoine, 2011). The ability to conclusively test face validity in genetic models is therefore vital if we wish to exclude models that are unlikely to provide a window onto human pathology (Garner, 2014).

Where genetic models fail to exhibit detectable spontaneous seizures, one recourse is to induce seizures using chemoconvulsants or auditory stimuli. By varying the auditory stimulus or chemoconvulsant dose, researchers can assess whether genetic mutants have increased seizure susceptibility compared with controls (De Sarro et al., 2004; Musumeci et al., 2000; Okuda et al., 2017; Pacey et al., 2009). This approach seems to marry the precision of genetic models with the utility of induced models in terms of seizure measurement. However, the fact remains that these models do not have seizures without the application of proconvulsant stimuli.

As discussed above, it is clear that the clinical community requires new ASDs with novel targets and mechanisms of action to address drug resistance in epilepsy. There is also a pressing need to develop disease-modifying anti-epileptogenic therapies that can reverse epileptogenesis rather than only providing seizure control (Baulac et al., 2015; Bialer et al., 2018; Pitkänen et al., 2013). If researchers can find ways to validate clinically relevant genetic models of epilepsy, we will have succeeded in capturing the pathophysiology associated with diverse epilepsy aetiologies. These models could then be used for the discovery of novel anti-seizure or anti-epileptogenic drugs.

To improve assessments of face validity in genetic models of epilepsy, researchers have continued to develop video-EEG seizure monitoring and are generating alternatives to EEG-based seizure detection. Continuous video-EEG monitoring is the gold-standard approach for characterising both genetic and induced rodent epilepsy models (Fig. 2), as it facilitates accurate and reliable seizure detection (Gu and Dalton, 2017). Over the past decade, there have been advances in the development of both the hardware and software used to capture and analyse rodent EEGs. Besides the open sharing of system designs and acquisition software, multi-channel electrophysiology amplifiers have decreased in price by orders of magnitude, enabling more research laboratories to rapidly and inexpensively implement recording systems (Hermiz et al., 2016; Siegle et al., 2017; Wasilczuk et al., 2016). Signals can be digitised directly on the mouse through advance head-stage amplifiers, thus negating artefacts caused by cable movement. Meanwhile, advances in microelectrode fabrication techniques allow high channel counts in a relatively small surface area, enabling the acquisition of EEGs with higher spatial resolution and brain coverage (Obien et al., 2014; Wasilczuk et al., 2016). Furthermore, multiple wireless recording systems are now available that allow long-term recordings without tethering mice, providing an opportunity for improved animal welfare (Chang et al., 2011; Jiang et al., 2017; Lidster et al., 2016).

As many epilepsy models have only rare seizures, researchers need to record and monitor animals for a period of weeks to months to establish an accurate seizure baseline (Gu and Dalton, 2017). To streamline the detection and quantification of EEG abnormalities over these timescales, numerous freely available open-source algorithms can automatically detect interictal (Box 1) spikes and/or seizures from rodent EEGs (Anjum et al., 2018; Bergstrom et al., 2013; Brown et al., 2018; Buteneers et al., 2011; Casillas-Espinosa et al., 2019; Colasante et al., 2020; Jang and Cho, 2019; Pfammatter et al., 2019; Tieng et al., 2016, 2017; Wykes et al., 2012), either offline or in real time. EEG traces typically contain artefacts caused by electromagnetic or biophysical interference (Kadam et al., 2017; Pfammatter et al., 2019), but many algorithms can automatically or semi-automatically filter these out (Anjum et al., 2018; Bergstrom et al., 2013; Buteneers et al., 2011; Casillas-Espinosa et al., 2019; Tieng et al., 2016). As a result, many of these systems report sensitivities and specificities approaching 100% for the detection of epileptiform EEG abnormalities (Anjum et al., 2018; Bergstrom et al., 2013; Buteneers et al., 2011; Casillas-Espinosa et al., 2019; Jang and Cho, 2019; Pfammatter et al., 2019; Tieng et al., 2016).

Automating EEG-based detection of seizures will allow researchers to overcome many of the challenges associated with validating genetic epilepsy models with problems of the first sort, including rare seizures or seizures that predominantly occur during certain stages of the sleep-wake or life cycles. Indeed, algorithms have successfully detected and tracked seizures occurring as rarely as 0.3 times per week (Anjum et al., 2018). Moreover, automated seizure classification could remove the subjectivity and human error inherent to manual EEG interpretation that often result in considerable variability (Abend et al., 2011; Duez et al., 2018; Halford et al., 2015).

Despite these technical advances, there remain issues relating to the use of EEG in rodents, especially long-term continuous EEG. The implantation of intracranial electrodes or installation of electrode arrays is an invasive and time-consuming surgical procedure, which itself could cause damage leading to epileptic activity (Burman and Parrish, 2018). Moreover, to prevent animal injury and damage to recording equipment, animals are often housed singly, both post-operatively and over the duration of continuous EEG recordings, which can last for months. Mice and rats are social animals and it is now well established that single housing causes stress (Ieraci et al., 2016; Lukkes et al., 2009; Manouze et al., 2019; Weintraub et al., 2010). Social isolation of young mice results not only in behavioural abnormalities but also increased seizure susceptibility in adulthood (Amiri et al., 2017). There is also evidence that social isolation can affect seizure phenotypes in adult male mice (Amiri et al., 2014; Matsumoto et al., 2003). Meanwhile, a recent study in rats showed that socially isolated animals had a 16-fold increase in spontaneous seizure frequency following treatment with the cholinergic agonist pilocarpine (Manouze et al., 2019).

**Fig. 3.**
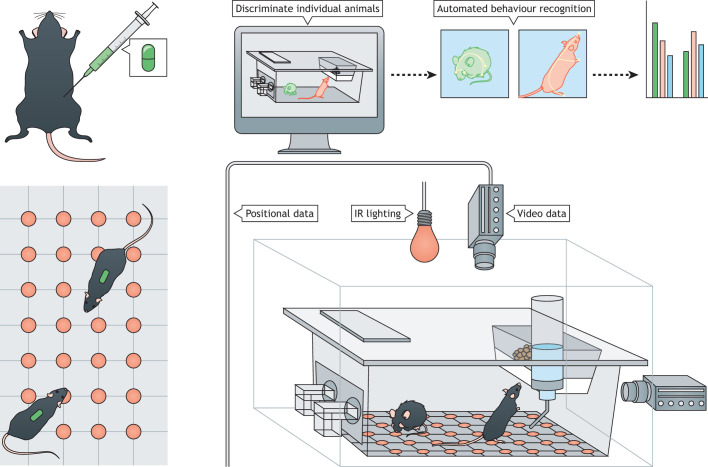
**RFID-assisted in-cage monitoring system.** Standard rodent cages are placed within the in-cage monitoring system, where cameras positioned above or at the side of the cage allow continuous video monitoring. Infra-red (IR) cameras and illumination are preferred as they allow animals to be visualised during the dark phase of their 24-h light/dark cycle (Bains et al., 2016; Gu and Dalton, 2017). The majority of in-cage monitoring systems can only monitor single animals (Bains et al., 2018). Increasingly, subcutaneous radio-frequency identification (RFID) chips (depicted as green tags in the figure) are being used to discriminate individual animals within groups (Bains et al., 2016; Krackow et al., 2010; Peleh et al., 2019). The position of several RFID-tagged animals can be continuously tracked as they move over an RFID antenna array (red dots) beneath the home cage and/or near areas such as water bottles or food hoppers.

Researchers need to develop accurate and reliable methods of seizure detection that are minimally invasive and that can be employed in group-housed rodents over extended time periods. In recent years, the most promising developments towards this goal have come from radio-frequency identification (RFID)-assisted in-cage monitoring systems (Fig. 3).

In-cage monitoring systems were developed to meet the need for continuous assessment of unforced behaviours in rodents. At present, the majority of these systems require single housing. However, a growing number of systems are capable of continuously monitoring individual animals within groups using subcutaneous RFID tags (Bains et al., 2016; Krackow et al., 2010; Peleh et al., 2019). Although there are currently no in-cage monitoring systems with validated seizure-detection capabilities, both continuous video capture and animal movement tracking present opportunities for non-invasive detection of overt seizures. In a recent study, Jankovic et al. (2019) employed a video-based movement tracking system called EthoVisionXT to continuously monitor animals following repeated injections of the convulsing agent pentylenetetrazole (Jankovic et al., 2019). The authors found that convulsive seizures (Box 1) were associated with brief spikes in whole-animal mobility and used these spikes to highlight sections of the recording to further screen for seizures. Although the study did not report the sensitivity and specificity of this approach, it established the potential for mobility-based seizure screening in rodents exhibiting the most overt seizures.

Aside from gross motion tracking, home-cage video monitoring could eventually enable the detection of behavioural seizures (Box 1) based on finer visual features. Machine-learning approaches have been used to generate video-based classifiers that can recognise and score a range of rodent behaviours in real time or offline. The repertoire of such detectable behaviours is constantly expanding and currently includes locomotion and climbing (Bains et al., 2018), eating and rearing (Aniszewska et al., 2014), and more subtle behaviours such as scratching (Park et al., 2019) and head bobbing (Baker, 2011). Video-based classifiers for seizures have not yet been developed, but many of the behaviours for which classifiers currently exist – such as rearing, scratching and head bobbing – are seen in rodent seizures (Kandratavicius et al., 2014) and could form the basis for future classifiers. The generation of video-based classifiers for overt seizures is likely to be labour intensive, requiring manual classification of many instances of a given seizure type (Sturman et al., 2020). However, once classifiers are developed for a given type of seizure, they could be used to characterise other models with similar seizure presentations.

Movement or video-based seizure detection would necessarily be limited to seizures with overt behavioural involvement. As discussed above, many rodent epilepsy models exhibit only electrographic or absence seizures, which will always require EEG for detection. Nevertheless, robust, non-invasive seizure monitoring would represent an important refinement for the subset of epilepsy models that exhibit overt seizures and that have already been characterised electrographically.

Even without seizure-detection capabilities, in-cage monitoring is a relevant approach in preclinical epilepsy research. There is a growing appreciation of epilepsy as a spectrum disorder including not only spontaneous and recurrent seizures, but also cognitive, social and behavioural deficits (Fisher et al., 2014; Greenberg and Subaran, 2011; Holmes and Noebels, 2016; Sirven, 2015). These deficits can be the sequelae of chronic seizures or epileptiform activity, i.e. epileptic encephalopathy, but can also arise in parallel with seizures as a result of genetic mutations. In-cage monitoring of group-housed animals offers the opportunity to study levels of activity and social interaction in an ethologically relevant context, unlocking new behavioural domains in which face-valid phenotypes might be present. This could enhance the translational value of genetic epilepsy models by allowing direct assessment of any reversal of social and behavioural deficits during preclinical trials.

## Conclusions

To make progress in finding new treatments for epilepsy, as with all disorders, researchers will need to develop a range of different model systems (Box 2) and use them in a rational, integrated way. Initial drug screening can be carried out cheaply and efficiently in model systems like zebrafish larvae, where specific genetic alterations can be induced using CRISPR gene editing. Convulsive swimming movements can be captured with video monitoring and scored, with larvae arrayed in microtitre plates in which different wells can be exposed to different drugs. This system allows for initial high-throughput, relatively cheap screening that can then be followed up with electrophysiological confirmation (Griffin et al., 2016). Candidate drugs from such screens can then be directly tested in appropriate mammalian models; ultimately, rational drug design may allow direct mammalian testing approaches.

Novel therapies for epilepsies, even for those not caused by mutations in a single gene, may also emerge from the fields of gene therapy/gene targeting. Conventional gene therapy, in which loss of function of a specific gene is corrected by delivery of a viral vector encoding the missing product, is already being tested in monogenic epilepsy models (e.g. Niibori et al., 2020). Further developments include the use of antisense oligonucleotides to upregulate expression of the sodium channel Na_v_1.1, leading to a reduction in seizure frequency and severity in a mouse model of Dravet syndrome (Han et al., 2020).

Additional novel approaches that target the underlying mechanisms rather than specific genes could allow wider populations to be treated. Recent striking examples include the testing of autoregulatory gene therapy, in which neurons are inhibited in response to rises in extracellular glutamate (Lieb et al., 2018) and the use of CRISPRa (which modulates promoters) to upregulate expression of the potassium channel gene *Kcna1* in excitatory neurons, dampening neuronal excitability and reducing seizure frequency in a mouse model (Colasante et al., 2020).

Thus, the use of multi-pronged strategies may also allow us eventually to circle back from the initial blunt approach of finding drugs that treated seizures with no regard to underlying cause, to finding that therapies initially designed for monogenic epilepsies may, in fact, benefit much broader patient groups on the basis of their underlying genetic susceptibilities. Heritability estimates based on twin studies support a strong genetic component in epilepsy overall (Kjeldsen et al., 2001; Miller et al., 1998), and up to 80% of epilepsy cases are attributable to genetics (Koeleman, 2018), with many considered to have polygenic inheritance patterns (Hildebrand et al., 2013; Koeleman, 2018). Furthermore, many genes mutated in severe early-onset genetic epilepsies have also been implicated in other types of epilepsy, either as milder mutations (Qaiser et al., 2020), as genetic modifiers (Mirza et al., 2017), or on the basis of network analysis showing overlap between genetic causes and drug targets (Delahaye-Duriez et al., 2016). In addition, it is becoming clear that diverse neurodevelopmental disorders converge onto common molecular pathways (Auerbach et al., 2011; Barnes et al., 2015). It is therefore possible that animal models of monogenic epilepsies may yield therapies with broader treatment applications.

If genetic models can lead to the development and testing of specific therapies aimed at monogenic epilepsies, the same drugs can then be tested in induced seizure models on the basis that altering the expression or function of target genes may have wider applicability. This can, in turn, benefit much broader patient populations and thus help to alleviate problems associated with epilepsy and other neurodevelopmental disorders in large numbers of individuals and their families.
